# Translating real‐world evidence/real‐world data

**DOI:** 10.1111/cts.13835

**Published:** 2024-05-16

**Authors:** Paulien Ravenstijn

**Affiliations:** ^1^ Astellas Pharma Leiden The Netherlands

The theme of the first Special Collection in *Clinical and Translational Science* is *Real‐World Evidence/Real‐World Data* (https://ascpt.onlinelibrary.wiley.com/doi/toc/10.1111/(ISSN)1752‐8062.RealWorld) and was chosen to complement the ASCPT Preconference Workshop on “Advancing the Utilization of Real‐World Data (RWD) and Real‐World Evidence (RWE) in Clinical Pharmacology and Translational Research” that took place on March 21, 2023. At ASCPT, the interest in the RWE/RWD topic was already expanding in 2021 when a cross‐network collaboration was set up in ASCPT to increase the knowledge around RWE/RWD in clinical pharmacology and translational science. At the time, it was felt that RWE/RWD was still a buzz‐word with limited knowledge on what it actually was and how it could be applied. The ASCPT cross‐network collaboration worked together with the IQ Consortium, a pharmaceutical and biotechnology association, to organize the Preconference Workshop mentioned above and include speakers covering the academic, industrial, and regulatory perspectives. Lui et al.^1^ describe the proceedings of this workshop that included four state‐of‐the‐art lectures, five case studies, and a hands‐on exercise (Table 1) where participants discussed opportunities for implementing RWD and RWE in the development of a hypothetical Drug X for the treatment of Duchenne muscular dystrophy. While it was agreed that the application of RWE/RWD is not commonly used in clinical pharmacology, translational research, drug development, and approval yet, it does have the potential to be implemented across all stages of development and for internal decision‐making when the right question is defined and taking its limitations into consideration. This special collection is meant to provide examples of the current application of RWE/RWD within clinical pharmacology and translational science.

**TABLE 1:** Key learnings from the hands‐on workshop on the potential opportunities of using RWD/RWE in drug development and approval of Drug X for treatment of DMD.^1^

Drug development question	Opportunities for RWE/RWD
Quantitative target product profile	Identify unmet medical needs and subpopulations (e.g., age groups may benefit from drug X) Inform quantitative differentiation criteria on efficacy/safety versus glucocorticoids (SOCs)
Translational research/Biomarker identification	Identify biomarkers that demonstrate dystrophin production Identify biomarker/DMD and biomarker/efficacy relationships, specifically regarding long‐term benefits Identify a biomarker for early disease and/or treatment effect read‐out Identify biomarkers that correlate with safety/toxicity
Disease progression	Characterize the natural history of DMD disease progression using longitudinal data and modeling and simulation to support clinical trial design and interpretation of trial results Understand the effects of disease onset time/stage on disease progression, biomarker and efficacy endpoints Identify more sensitive endpoints to predict DMD disease progression and long‐term efficacy Complement clinical trial data
Clinical trial design	Leverage modeling and simulation to establish external/synthetic control Include suitable and diverse patient populations Incorporate sensitive and novel endpoints for efficacy and safety Design adequate trial duration based on learnings from disease progression Incorporate more realistic inclusion/exclusion criteria informed by RWD based DDI liability assessment
Clinical DDI (glucuronidation & CYP3A metabolism, moderate inducer of CYP3A4)	Identify concomitant medication use and the potential DDI liability, specifically regarding CYP inducers Establish PBPK model using probes identified from RWD and Phase II data to identify the magnitude of interaction Identify which drug metabolism enzymes/transporters should be addressed for DDI early on in drug development
Special populations	Identify prevalence of renal impairment and hepatic impairment in DMD to inform clinical trial inclusion/exclusion criteria and need for conducting the organ impairment studies Identify alternative marker for renal function in DMD as creatinine may be confounded with muscle dystrophy Inform pediatric patient age group to be studied Generate evidence for extrapolation from older to younger pediatric patients if supported by RWE from SOCs

Abbreviations: CYP, cytochrome P450; DDI, drug–drug interaciotn; DMD, Duchenne muscular dystrophy; PBPK, physiologically‐based pharmacokinetic; RWD, real‐world data; RWE, real‐world evidence; SOC, standard of care.

Applying RWE and RWD in supporting regulatory decision‐making may not be straightforward. Yuan et al.^2^ compared two prominent publications on the role of RWD and RWE in the drug approval process. They identified similarities and differences regarding categorization of the use of RWE to support drug approvals and hence showed that there may be a need to develop a consistent understanding among regulatory agencies and stakeholders.

One of the publications by Dagenais et al.^3^ that is included in this special collection proposes the use of RWD of a target patient population to evaluate the benefit/risk profile of drug candidates that show drug–drug interactions (DDI). The study described here included RWD on CYP3A, CYP2D6, or CYP2C9 substrates and patients suffering from either Type 2 diabetes mellitus, obesity, or migraine. It serves as an excellent example of how the predicted DDI impact can help in comparing different drug candidates and possibly favor the candidate with the lowest DDI liability.

Real‐world data from a cancer patient population that underwent *DPYD* and *UGT1A1* pharmacogenetic testing were the subject for the paper by Muldoon et al.^4^ The objective of the retrospective analysis of these RWD was to demonstrate the feasibility of the *DPYD* and *UGT1A1* pharmacogenetic testing prior to starting chemotherapy in these patients, specifically for community‐based cancer centers. Operationally, it was shown that the majority of the pharmacogenomic results were available and interpreted before chemotherapy initiation. The study was also able to investigate the number of actionable opportunities (i.e., dose reductions) related to *DPYD* and *UGT1A1* outcome, and if they were implemented before or after start of chemotherapy as well as unplanned hospitalizations and treatment interruptions in relation to different genetic variants.

On a similar note, Ramste et al.^5^ used RWD on purchased prescription drugs, hospital discharge, death, and genotype for CYP2C19*2, CYP2C19*3, and CYP2C19*8 loss‐of‐function (LOF) variants from a cohort of prospective patients with acute coronary syndrome or symptomatic chronic coronary disease. The authors observed significantly more cardiovascular events in carriers of *CYP2C19* LOF variants and in non‐carriers using the CYP2C19 inhibitor omeprazole or esomeprazole. It was therefore concluded that both genetics and concomitant medications should be considered for optimal patient care.

Tacrolimus is an agent with a narrow therapeutic range and is metabolized by CYP3A4/5. Alghamdi et al.^6^ analyzed electronic health records to investigate the effect of CYP3A5 metabolism (intermediate and normal metabolizers [M/NM] versus poor metabolizers [PM]) on reaching the Tacrolimus therapeutic range. CYP3A5 IM/NM took longer than PM to reach the therapeutic range, had more dose adjustments, and needed >150% of the required daily dose compared with PM. They concluded that preemptive genotyping could decrease the number of dose changes necessary to reach a therapeutic dose.

Another example of how analysis of RWD can be used to adjust a treatment approach and optimize therapeutic outcome is described by Kim et al.^7^ They used RWD from electronic health records to identify BMI ≥23 kg/m^2^, the use of proton pump inhibitors, cholinergic medications, and the presence of cardiovascular diseases as risk factors for hypoglycemic adverse events in patients diagnosed with coronavirus disease 2019 (COVID‐19) who are treated with remdesivir. They developed a risk‐scoring system that enables early identification of patients at risk of hyperglycemia during remdesivir treatment.

Mandema et al.^8^ performed a retrospective comparison of real‐world studies (RWS) and randomized controlled trials (RCTs) from eight treatment regimens for COVID‐19 to determine a rapid an robust strategy for drug repurposing in future pandemics. The comparison showed that the focus should be on large well‐conducted RCTs. Additionally, improvements to reporting of RWS and utilization of bias‐reducing methodologies may improve their integration and utility as part of a holistic evidence‐based assessment.

The second publication in the special collection from Dagenais et al.^9^ shows how RWD from a large and heterogeneous patient population across the full age spectrum can be used for the estimation of the differences in glomerular filtration rate (GFR) and/or creatinine clearance (CrCl) change between the current reference standard equation in each age stratum and other commonly used equations. As currently multiple equations are used for the estimation of GFR and/or CrCl across patients populations and age ranges, this RWD study may be a first step in the implementation of one or two equations across the full age spectrum.

Finally, Kou et al.^10^ developed and validated a risk prediction model for sepsis among patients with liver cirrhosis using data from the Medical Information Mart for Intensive Care IV database. Multivariate logistic regression was used to establish the prediction model and was then validated.

The first special collection in *Clinical and Translational Science* focuses on RWE/RWD and complements the ASCPT Preconference Workshop on this topic. The application of RWE/RWD is not commonly used in clinical pharmacology and translational research yet, but it has the potential to be implemented across all stages of development, regulatory approval, post‐marketing, and for internal decision‐making. In conclusion, this collection aims to spark interest in the application of RWE/RWD in the fields of clinical pharmacology and translational science. Hopefully, it encourages readers to explore the potential of RWE/RWD in their future research endeavors.

## FUNDING INFORMATION

No funding was received for this work.

## CONFLICT OF INTEREST STATEMENT

The author declares no competing interests for this work.
